# Peer reviewers’ dilemmas: a qualitative exploration of decisional conflict in the evaluation of grant applications in the medical humanities and social sciences

**DOI:** 10.1057/s41599-022-01050-6

**Published:** 2022-03-04

**Authors:** Gaёlle Vallée-Tourangeau, Ana Wheelock, Tushna Vandrevala, Priscilla Harries

**Affiliations:** 1 Kingston Business School, Kingston University, Kingston upon Thames, UK; 2 Centre for Applied Health and Social Care Research, Kingston University and St. George’s, University of London, London, UK

## Abstract

Independent evaluations of grant applications by subject experts are an important part of the peer-review system. However, little is known about the real-time experiences of peer reviewers or experts who perform reviews of a grant application independently. This study sought to gain insight into this stage of the grant evaluation process by observing how experts conduct an independent review in near real time. Using the think aloud approach and Critical Decision Method of interviewing, in-depth interviews were conducted with 16 peer reviewers from a range of roles and disciplines within the medical humanities and social sciences. Participants were asked to think aloud while reviewing applications to different grant schemes from a single prestigious funder. The analysis shows reviewers encountered five dilemmas during the evaluation process. These dilemmas were related to whether or not one should (1) accept an invitation to review, (2) rely exclusively on the information presented in the application, (3) pay attention to institutional prestige, (4) offer comments about aspects that are not directly related to academics’ area of expertise, and (5) to take risks and overlook shortcomings rather than err on the side of caution. In order to decide on the appropriate course of action, reviewers often engaged in a series of deliberations and trade-offs—varying in length and complexity. However, their interpretation of what was ‘right’ was influenced by their values, preferences and experiences, but also by relevant norms and their understanding of the funder’s guidelines and priorities. As a result, the way reviewers approached the identified dilemmas was idiosyncratic and sometimes diametrically opposed to other reviewers’ views, which could lead to variation in peer-review outcomes. The dilemmas we have uncovered suggest that peer reviewers engage in thoughtful considerations during the peer-review process. We should, therefore, be wary of reducing the absence of consensus as resulting from biased, instinctive thinking. Rather, these findings highlight the diversity of values, priorities and habits and ways of working each reviewer brings to the fore when reviewing the applicants and their project proposals and call for further reflection on, and study of, this “invisible work” to better understand and continue to improve the peer-reviewing process.

## Introduction

Research funders often rely on scientific experts (peer reviewers) to evaluate the quality of grant applications from researchers working in the same or similar fields. The peerreview process is typically comprised of three stages: external or independent review by subject experts, internal review by grant funding panel members, and research panel discussions (Abdoul et al., 2012). Independent review is usually conducted separately by two or more subject experts. Their judgements are intended to support research panel’s internal reviewers to identify outstanding applications and are generally in line with funding decisions (Guthrie et al., 2017). Research panel members use independent reviewers’ recommendations as a basis for discussion, and consensus is reached on which projects to put forward for funding. Sometimes shortlisted applicants are interviewed by the panel as part of the review process.

The legitimacy of the grant peer-review process relies on the premise that peer assessment protects the autonomy of the academy and ensures that research funding decisions are based on merit (Feller, 2013). However, inconsistencies in experts’ evaluations and declining success rates have spurred the scientific community to scrutinise the mechanisms used to allocate research funding.

Critics claim peer review is “broken” and, in its current form, unable to fulfil its primary aim, which is to identify the best grant applications for funding. They argue that peer-review processes are unfair and often biased against female investigators (Kaatz et al., 2016; Witteman et al., 2019), interdisciplinary research (Feller, 2006; Travis and Collins, 1991) and applicants who are unknown or disagreeable to reviewers (Abdoul et al., 2012; Marsh et al., 2007). Another target of criticism is the lack of reliability of reviewers’ evaluations, particularly if they are inexperienced (Gallo et al., 2016; Jayasinghe et al., 2003). Peer review has also been criticised for stifling innovation (Cole et al., 1977; Greenberg, 1998), being inefficient and burdensome, especially for applicants (Barnett et al., 2015; Gluckman, 2012), and incapable of predicting success (Danthi et al., 2014; van den Besselaar and Sandström, 2015). These shortcomings have fuelled efforts to explore alternative funding processes to improve the trustworthiness, fairness and efficiency of peer review, such as random application selection by lottery (Avin, 2019).

Evidence on some of these failings, however, are methodologically heterogeneous and inconclusive (Guthrie et al., 2017; Lee et al., 2013) and much of these criticisms stem from studies investigating associations between aspects pertaining to applicants or applications and funding recommendations (Lee et al., 2013). While these studies may reveal important deficiencies and limitations of peer review, they shed little light on how reviewers’ judgements are shaped and what influences their choices. This lack of understanding can lead to oversimplified recommendations and a tendency to apportion excessive blame on reviewers for the ills of the peer-review system.

Qualitative studies on research panels by Lamont and colleagues have contributed to addressing this knowledge gap by examining grant evaluation as is, as opposed to how it ‘should’ be. They show that peer review is deeply influenced by contextual, psychological and social considerations (Lamont, 2009; Mallard et al., 2009) and characterised by significant tensions, notably those between application appraisal and self-interest, and between democratic values (e.g., consensus) and expertise (Lamont, 2009). This research also reveals that panel members conceive fair decision-making not as one that uses generalisable review criteria, as advocated by a number of scholars who equate fairness in peer review to adherence to the norm of universalism (Collins and Evans, 2002; Merton, 1973), but one that uses the most appropriate epistemological style to the discipline of the application under evaluation (Mallard et al., 2009). One of the most important contributions of Lamont and her collaborators is drawing attention to peer review as an inherently human endeavour, which is carried out by “emotional, cognitive and social beings who necessarily interact with the world through specific frames, narratives and conventions, but who nevertheless develop expert views concerning what defines legitimate and illegitimate assessments, as well as excellent and less stellar research” (Lamont and Huutoniemi, 2011, p. 47). This work also underscores how qualitative research, and observational studies in particular, can help us to develop a more contextualised and accurate understanding of peer review.

Furthermore, there is an important distinction research can draw to advance our understanding of the grant peer-reviewing process: namely, that different contexts in which peer review activities take place may involve specific psychological and behavioural processes. While all stages of the grant peer review process share similarities, it is important to highlight key differences, which may play a unique role in the overall review outcome.

For example, Abdoul et al. (2012) noted that external expert reviewers spent a considerable amount of time reviewing a proposal (from a few hours to several days) and may search for previous studies on a topic. Internal reviewers sitting on funding panels, by contrast, usually spent a couple of hours at most reviewing the proposal, heavily relied on the reviews written by external expert reviewers, and rarely if ever searched the literature in preparation for the panel discussion. During panel deliberations, the time available to review each proposal was even shorter, and discussion times were noted to vary as a function of the eloquence of the internal reviewer, whose chief task was to present a synthesis of the evaluations.

Although research on decision-making suggests there are some similarities between group and individual decisions, they are often weakly related to each other; while individual decisions are more influenced by biases, cognitive limitations and social considerations than group decisions (Charness and Sutter, 2012), the latter are vulnerable to peer pressure and groupthink (Esser, 1998; Olbrecht and Bornmann, 2010). Given the importance of the recommendations of independent reviewers in the award of research funding, the purpose of this study is to examine how the evaluation of grant applications unfolds in the first stage of the grant peer-review process, when individual external reviewers carry out their evaluations.

This study forms part of the TORR (Towards Outstanding Research Reviews) project, a proof-of-principle project on independent peer review in the medical humanities and social sciences. This research is the first to observe how peer reviewers conduct a review of a grant application in near real time, rather than relying on their interpretation of their decision-making processes. In the study reported here, we aim to gain insight into the experience of individual experts, from a phenomenological standpoint. Specifically, we seek to shed light on “the horizons and background assumptions” (Moran, 2000) peer reviewers bring about during the act of thinking about a grant application. The concept of horizon in phenomenology refers to the proposal that perception is informed both by what we perceive and what we expect to perceive. Relatedly, background assumptions refer to the implicit pre-judgements we bring with us when we seek to understand and interpret our experiences. In this study, the “horizons” of the expert reviewers are the possibilities they may perceive or anticipate while reviewing the grant application, which go beyond the information contained explicitly in the written application document. Similarly, their “background assumptions” refer to their unique implicit pre-judgements, which may impact their experience and interpretation of the information presented in the grant application they are reviewing.

## Methods

### Design and theoretical underpinning

To explore in-depth individuals’ phenomenological experience of the grant reviewing process aiming to describe their thoughts as they appear to consciousness, a qualitative approach was considered appropriate (Malterud, 2001). Of particular importance is the *Big Q* approach to qualitative work, that is, an approach within a qualitative paradigm that does not seek to quantify data and do not aspire to an *objectivity* that is problematised, but instead works with the interplay of researchers and participants’ subjectivities (Kidder and Fine, 1987).

Our data collection approach drew from the think aloud method (Svenson, 1979; van Someren et al., 1994) and the Critical Decision Method of interviewing (CDM) (Klein et al., 1989). Briefly, the think aloud method was designed to understand problem-solving processes by asking people to think aloud while they are engaged in a cognitive task analysing the resulting thought-listing or ‘verbal protocol’ (van Someren et al., 1994) and can be particularly useful to understand the reasoning behind experts’ decision-making processes. Verbal protocols can be simultaneous (when participants say aloud any thought that comes up while working on a task) or retrospective (when participants describe their decision-making process once the task has been completed) (Svenson, 1979). The CDM is a retrospective interview method that applies a set of cognitive probes to real non-routine incidents requiring expert judgement (Klein et al., 1989). It posits that more detailed and actionable information is obtained when concrete events are examined than when general rules and processes are elicited.

Our research design combined both verbal protocol approaches, by asking participants to provide a simultaneous report of their review process of a grant application they had recently evaluated, while relying on recall to condense in a short period of time a review that would normally take three hours or more. The CDM procedures were also adapted to suit the peerreview process. Specifically, we excluded the construction of the ‘event’ timeline, because peer review is seldom a linear process.

We used a semi-structured and probing interview schedule, informed by a review of the literature and our team’s expertise. Based on the CDM methodology, we employed a think aloud approach combined with more traditional eliciting techniques to reach a comprehensive understanding of the “live” peer-review process.

The interview schedule followed a 3-step approach. Firstly, we asked participants to provide an unstructured account of the review process they had undertaken, which provided us with unadulterated context and a phenomenological perspective of the participant’s trajectory. Secondly, participants were asked to read and comment on a specific grant application they had recently reviewed using the think aloud approach, as if they were assessing it for the first time, and salient decisions points or pivots were recorded. Thirdly, participants provided a general appraisal of the application and review process and unclear points were elucidated. Key participant socio-demographic information was collected at the end of the interview, including their age, gender, current role, discipline and experience evaluating research proposals.

We opted for this method rather than a more traditional qualitative interview approach to enable peer reviewers to share their experience freely and to reduce the possible influence of social desirability bias or selective recall on their responses. We reasoned that a retrospective interview may have limited the richness of the peer reviewers’ account of their experience as they would have had to rely on their memory and imagination. By contrast, we anticipated that revisiting a grant proposal they had recently reviewed would provide a better platform to cue their recall of their actual experience of peer reviewing. The think aloud method allows documenting cognitive processes while they are taking place. We favoured this approach to increase the likelihood of capturing detail and spontaneous thoughts and feelings about the different elements of the proposal (e.g., about the applicant, the supporting institution, etc.), but also (and most crucially for our present purpose) about their own role as reviewers.

### Sampling and recruitment

Since the research team’s academic expertise falls within the remit of medical humanities and social sciences, this study focused on peer review of grant applications in this broad area. Following consultation with the Wellcome Trust (henceforth ‘the funder’), we identified their Awards in Humanities and Social Science as viable targets. We aimed to purposively recruit 15 to 20 reviewers, an acceptable sample size to reach data saturation (Patton, 2005). To reduce recall bias, we first invited reviewers who submitted evaluations to the most recent funding round. To increase our sample size, we then invited reviewers who evaluated applications to the previous round.

A total of 44 scholars who had reviewed grant applications for the July 2018 and January 2019 rounds of the Wellcome Trust Awards in Humanities and Social Science were invited to participate and 16 reviewers were interviewed between July and December 2019 (36%), a response rate mirroring that of other studies using similar populations (Dykema et al., 2013). The characteristics of participants (*N* = 16) and the overall population (*N* = 44) are presented in Table 1.

### Overview of the funder’s peer-review procedure

Typically, the funder sends experts an invitation to review, which includes the name and institution of the applicant, the title of the project and a link to a digital review platform where they can access the full application form. Reviewers are asked to evaluate the application and comment on key aspects (depending on the type of award), such as the applicant, the research environment, the proposed research, outputs management and sharing, and resources. They are also asked to provide a rating reflecting their opinion of the overall merit of the proposal.

### Study procedure

Once reviewers had submitted their evaluation, the funder emailed potential participants an invitation, on behalf of the research team, to take part in our research, as well as the study’s information sheet and consent form. The invitation letter introduced the project and provided a summary of the interview process. Upon agreement to participate, the funder uploaded the reviewers’ signed consent forms and contact details, the research proposal they had evaluated and their written review onto a secure data sharing platform. Our research did not interfere at any point with the funding decisions of the grant review panels regarding the evaluated proposals.

Reviewers were then contacted by our team via email to arrange an interview and were given an opportunity to ask questions and were informed of their rights to anonymity, confidentiality and withdrawal. The interviews were conducted remotely using the Zoom application. Permission was sought for the interview to be recorded (screencast video and audio). Subsequently, the interviewer (AW) shared an abstract of a generic grant application for participants to practice thinking aloud and then, once comfortable with the approach, a copy of the grant application the participant had previously assessed alongside their written review. Participants were given remote control of each document in turn, so they could read them as if they were working from their own computer. Participants were then asked to think aloud while assessing the documents as if they were ‘talking to themselves’.

The interview lasted approximately 60 min. Upon completion, participants were sent a debriefing sheet and given the opportunity to ask any outstanding questions. They were also given a £45 voucher as a token of appreciation for their time. Interviews were transcribed verbatim.

### Analysis

Interview transcripts were analysed using the online application Dedoose. A six-phase method suggested by Braun and Clarke was used to guide the analysis (Braun and Clarke, 2006). The phases include familiarisation with the data, generation of initial codes, search for themes, review of the potential themes, naming the themes and producing the report. We pursued a combined approach of inductive and deductive analysis to discover new information and identify themes aligning with theoretical propositions (Patton, 2002). The inductive approach was primarily used to explore the data and identify patterns and reoccurring themes. This approach allows the theory to emerge from the data (Thomas, 2006) and is particularly valuable when a new perspective is used to understand a problem. The deductive element of the analysis meant that it was guided by the research aim; analytic attention was paid to the phenomenology of participants’ motivations, experiences, perceptions and practices regarding the review process.

AW and TV familiarised themselves with the data. AW coded all the transcripts, and TV coded a sub-sample of the dataset independently. Emerging themes were identified by both researchers separately and differences were resolved through discussion and review until consensus was reached. Discussions between AW, TV, and GVT informed the latter stages of the analysis, with AW taking primary responsibility.

It is important to acknowledge the active role played by the research team in defining the themes reported in the next section (Braun and Clarke, 2006). We engaged with the data and met on several occasions to select, discuss, and refine the themes, which were deemed to be shedding significant light on peer reviewers’ experience of the reviewing process. In the excerpts presented to illustrate themes, participants’ names and other information that may identify them have been replaced by pseudonyms to protect confidentiality. The quality of the analysis was ensured through close alignment with recognised criteria for good qualitative research, such as grounding interpretations in examples from the data (which allows readers to confirm or query interpretations), conducting credibility checks and optimising coherence across the study (Elliott and Timulak, 2005; Yardley, 2000).

## Results

Participants’ trajectories suggest they were confronted with five distinct but inter-related considerations when evaluating grant applications. These considerations were non-linear and dilemmatic in nature. They were not simple or heuristic choices. Rather, they were thoughtful and often difficult decision points that illustrated a dimension of peer-reviewing practice that has remained unacknowledged and poorly evidenced, namely the moral conflict and social considerations at play in this otherwise voluntary, and often criticised professional activity. We present the evidence for these dilemmas before discussing them in the next section.

The five dilemmas included whether: (1) to accept an invitation to review, (2) to rely exclusively on the information presented in the application, (3) to pay attention to institutional prestige, (4) to offer comments about aspects that are not directly related to academics’ area of expertise, and (5) to take risks and overlook shortcomings rather than err on the side of caution.

### To accept an invitation to review

When evaluating whether to accept an invitation to review a grant application, participants’ accounts indicate they considered both the costs and benefits of doing so. Although the match between the topic of the project and their expertise was a salient aspect, some participants said they were more likely to accept a review if they were interested in the topic, were familiar with the work of the applicant(s) or mentor(s), believed in the intrinsic value of the project and/or felt that the research being proposed could benefit their own research.

I was interested in the project because I know the applicant. I’ve met [them] at a number of conferences, so I’m interested in [their] work. I’m very well aware of [their] area of study. So, I was initially prepared to accept the invitation to review it. It was interesting to me because anything that [they] found… would have an impact on my own research. (P5)

Gratitude toward the funder was mentioned by a participant as an important influence in their decision to accept a request to review.

…I generally have a policy of accepting requests from the [funder] because they have funded me quite generously in the past and I’ve still got a bit of funding at the moment. (P3)

Although having enough time appeared to be a general consideration for taking on a review, as indicated by participant 15, there was no mention of having rejected a review request from the evaluated funder on that basis.

…[peer-review] adds to my workload. So, my personal evaluation is: do I take this on or don’t I take this on? It depends on what workload I have at the moment… then I look at the deadline… very often, not the [funder], but other people… come up with what I think are preposterously ridiculous deadlines… (P15)

The act of peer reviewing appears to be dilemmatic from the onset, starting with the decision to accept or reject an invitation to review. Here, the dilemma is about dedicating time to a task that mostly benefits the collective (peer review) or spending it on activities that may be more advantageous to the individual (e.g., writing grants).

### To rely exclusively on the information presented in the application

There was a clear conflict between participants’ need to make informed recommendations and the appropriateness of accessing further information, either by using their prior knowledge of applicants or researching them using a search engine such as Google. Some participants were concerned that knowing the applicants would unduly influence their evaluation.

…I feel slightly ill at ease in knowing how much to deploy a kind of general understanding of the publication capabilities of people with the CVs that they have versus the words on the paper, because my worry… is that if I judge this application more favourably than the words, but then the next application I get I don’t know the people at all, then I’m prejudiced against that latter application. (P6)

Others noted that due to the expertise required to review a grant application, it was almost impossible not to know people in their fields, and that this knowledge could lead to more informed decisions about the quality of applicants.

I thought that the best strategy for me to adopt here was to just be upfront about my knowledge of the PI… I sort of in a way thought that I can use the conflict of interest or whatever bit of the submission… [to] summarise what my catch has been, but also just sort of… communicate quite clearly that that knowledge… also gives me the sense that the lead PI is in my view high quality. (P9)

Participant 9 was aware that their previous knowledge of the applicant may constitute conflict of interest but did not necessarily feel this could bias their evaluation. Instead, this participant believed that knowing the applicant and their work gave them a unique insight into their capability to deliver on the grant.

In one case, however, prior knowledge of the applicants and their research group appeared to negatively influence the participant’s judgements about the application.

…it’s a grant that’s coming out of [institution] again and I have a slight heart-sink feeling because… it’s a group which, does a very good job of pulling students through, but their students don’t seem to go anywhere… the whole group itself has got a real problem engaging with the wider community… I think [the mentor] is brilliant… an incredible powerhouse… I’m actually reading [the mentor’s section] to catch-up on what work [the mentor] is doing… I hadn’t realised that [the mentor] has written this [describes work and topic] and I’m thinking, well actually, I’m a [area of expertise], maybe I should have done that… I’m also really interested to see the size and, um, who the funders are of [the mentor’s] recent awards… I knew that everybody had, in like the community, had talked about how big the, um, [funder’s] award to [mentor’s group] was… but I think gosh… (P3)

Participant 3 expressed ambivalent feelings toward the applicants and their group, which often dominated their account. On one hand, it is clear the participant held the mentor and their work in high regard. On the other hand, disapproval of the group’s perceived insularity, feelings of regret for not having pursued similar research and emphasis on the size of the group’s research awards could signal implicit feelings of envy.

Notifying the funder about their knowledge of or links with applicants and trying to remain impartial was mentioned by some participants as a strategy to deal with potential conflicts of interest. Other participants routinely skipped the sections about the applicants and research environment and read the research project first – regardless of whether the applicants were known to them –, to reduce the risk of bias against or in favour of the application. One participant commented on how the ordering of the sections in the application form hindered their efforts to remain fair, and what this ordering beckoned regarding what they should pay attention to.

The research proposal comes quite late, so it kind of prioritises the candidature, the research environment, the reference letters more, above the actual proposal itself. And it tends to get hidden. For me, the proposal should be first and then you back it up with other stuff. It’s almost too much of window dressing before the proposal itself… by the time we reach the proposal on page 11 or 20 or whatever… Either you are exhausted or you have been persuaded by other ways that there’s a brilliant or not so brilliant proposal before reading the actual proposal itself. (P14)

In contrast, experienced reviewers who perhaps considered themselves to have a more secure position, felt it was easier to remain neutral towards competitors. As acknowledged by participant 4, senior researchers appeared to be more able to distance themselves from the work they were tasked with evaluating.

…these days I’m finding it a bit easier to distance myself from the involvement I may or may not have with a particular colleague, and the main reason for that is probably that I am, you know, I am almost [age], so I don’t have all that many career plans for myself, so I would not be reviewing competitors or very rarely actually these days and that will be for very, very big grants for which I apply, and that makes it a bit easier these days, because well, in the old days you know, when everybody is desperately trying to make a career 15, 20 years ago, then the kind of compartmentalising is more difficult and that’s why I think one has to be very cautious. (P4)

When applicants or their research environment were unknown to participants, a common approach was to seek more information using search engines, such as Google. While some participants gave some consideration to the appropriateness of researching applicants by contrasting it with a comparable activity (e.g., reviewing for journals), as illustrated by participant 6, others, such as participant 16, appeared to regard these searches as part and parcel of grant peer review.

The thing that I would always do is I would always Google the main collaborators in a research application… when it comes to reviewing for journals, even if you can work out who they are, it’s really bad form to have a look at who they are, to look at anything, you’re supposed to be just reviewing the paper, but I don’t take reviewing funding applications in the same way as I take reviewing a paper, so I would Google them, I’d have a look, and I would discover such things as there’s a big project going on at [institution]. (P6)

I would probably go on the web because I don’t know this person… I want to check who this person is [supervisor]. I want to check the supervisor, what they’ve done… (P16)

One participant, however, was expressly against researching applicants, because they considered it unfair and inappropriate.

I didn’t kind of like Google [them] and find out who [they were], because I felt that’s not appropriate for me to do, because I’m meant to be as unbiased as possible… I’m very old-fashioned, I meant to be genuinely reviewing [them] on the merits of the project that [they are] proposing. (P15)

In this case, the tension is between trying to remain impartial by focusing solely on the information presented in the application and supplementing this information with pre-existing knowledge (when applicants were familiar) or new information (when applicants were unknown).

### To pay attention to institutional prestige

Participants’ reports suggest their attitudes towards prestigious institutions were ambivalent. On one hand they noticed, and some explicitly valued, applicants who had been awarded grants from a recognised organisation, such as the funder, or had links with elite universities.

[The mentor] has attracted huge amounts of [the funder’s] funding and so there’s an awful lot of people in the field out there who are already connected to [the mentor]. (P3)

I was interested in the reference from [Professor] of [elite university], and obviously I read that carefully, that was the external examiner for the PhD dissertation. (P7)

On the other hand, some participants remarked that elite universities were excessively favoured by funders. Feelings of frustration were evident when the inclusion of an applicant or letter of recommendation from a recognised institution appeared to have little purpose other than to “bring shine” to an application.

…I think less deference to [elite university] would’ve made me feel a bit more positive, somehow there is just a rather unnecessary deference to [elite university] going on here. (P9)

Similar feelings where expressed by a participant about the inclusion of previous awardees of the funder as collaborators or referees, which was interpreted as being prompted by sections within the application form signalling the importance of being part of the funder’s “club”.

…the thing that irritates me is that the [funder’s] application is so much about recommendations from people and [funder’s] networks of funding… So, it’s got a different kind of feel. This one is, some of these boxes are kind of saying: are you one of us?… the danger of the “one of us” criteria that this form builds in is, from my point of view, it can actually, kind of put you off the application… they put in [funder’s] grants on the sponsor. And then there’s an enormous list on [funder-related] stuff. (P1)

Other participants went further and revealed they were particularly supportive of applicants from less known or well-resourced institutions as a strategy to introduce fairness into the funding system.

Do I mention the fact it’s in [institution’s city], and that [funder] is biased against the [region], and I mentioned that explicitly? No, I haven’t, but that’s in my head. And I would support people in non-prestigious organisations if their proposal is good and if they’ve got good support. Very happy to move money away from the expensive, posh, prestigious institutions towards less prestigious institutions and towards the [region], so I’m in favour of that in general, although, excellence is still my watchword on all this. (P10)

The conflict faced by participants was whether to take into consideration indirect evidence of future performance—such as prestigious recommendations or a track-record of grant awards— or to ignore this information as an attempt to level the playing field for excellent applicants from non-elite institutions or less experienced investigators.

### To offer comments about aspects that are not directly related to academics’ area of expertise

At times, participants also appeared to feel obliged to offer advice, even when they considered themselves ill-equipped to do so. For instance, multidisciplinary applications were considered important, albeit difficult to evaluate. Some participants noted that multidisciplinary research was more complex; hence, it required more work and focus. One participant suggested that reviewers needed to accept they would only be able to evaluate aspects that fell within their remit, rather than attempting to comment on aspects that did not.

And the fact that it’s a multidisciplinary approach interests me greatly, because I think there should be multidisciplinary activity among researchers. And it’s very, very difficult to get peer reviewers to take it seriously because, as I mentioned before, they only know one part of the project and can’t possibly, by definition, know all the parts and so they just have to be open to saying what they say about their bit and accepting that other people will comment on other bits. (P5)

Another case in point was when the budget was regarded by participants as extending beyond their area of expertise. In this case, the approach taken was to check whether anything was disproportionally costed.

I tend to ignore—or not ignore, but I place less emphasis on the money side of things, how much the people are applying [for]. So, the quantitative side is something that, frankly, I think is not any of my concern unless somebody asks for some really ridiculous sums of money for what is ostensibly a very small project. (P15)

The section on public engagement was also problematic. While some participants mentioned they paid attention to it because it was important to the funder, others appeared to feel uncomfortable about having to evaluate public engagement initiatives or questioned their appropriateness and need. Overall, weaknesses or absence of public engagement in applications were glossed over.

…so something that I wouldn’t necessarily tell the [funder] directly is that I think there’s a lot of nonsense about public engagement… I’m rather cynical about how much public engagement by professional academics really works… I’m not sure if it makes any sense that high ranking academics both do the research and then do the public engagement… I’m not sure why we insist on that, as long as there’s a publication strategy, then work gets out, but I’m not sure that running workshops for 10 people here and 10 people there is necessarily such a good thing. (P6)

The dilemma encountered by participants was whether to make a judgement for aspects that they might not consider to be directly related to their area of expertise or to withhold comment.

### To take risks and overlook shortcomings rather than err on the side of caution

Some participants were hesitant and at times conflicted about their decisions, particularly when certain aspects of the research proposal were outstanding and others substandard. One approach used to deal with these inconsistencies, especially when applicants were known to participants, was to focus on the strengths of the application and to either overlook problematic areas or to defer them to the panel for further enquiry at the interview stage.

I didn’t think that the governance for it was particularly well specified and for these interdisciplinary projects I think governance is very important actually, but I think partly because I know the principal applicant and the team and I just think that this is an interesting regional area and that… I know [the applicant is] good quality, I kept coming back to it and try to sort of read between the lines… (P9)

A contrasting approach was placing emphasis on the feasibility of the proposed research and being less ready to give participants the opportunity to amend weaknesses.

I thought it was a terrifically exciting project and really interesting questions… [But] I seem to remember having this vision of somebody being swamped with data and being all at sea and wondering what to do with it, which is often a problem, I think, in applications… So, I mean, in some ways, maybe I was a bit harsh… but I was worried because, yeah, I didn’t know that they were going to deliver on it. I mean in some ways, it would’ve been nice to go back to the person and ask them to redo that bit… (P12)

Reasons for taking a more cautious approach were a sense of duty to the funder and ensuring value for money, especially if the requested funding was high.

…the other thing that I think when I’m acting as a reviewer for funding agents, what I’m really thinking about, peculiarly, is I sort of think of myself as a kind of civil servant so I’m thinking of what will happen to the money, will the money be well spent and will there be outcomes? And so, what I do is I do a sort of bit of notional risks analysis… if the project had been for much less money, I’d have said, “Oh, yeah, fine” … we owe a duty of care to the funding organisation to be assured that something will come of all this, and it’s the [applicants] who reassure me primarily about that. (P6)

Taking risks, however, was not always frowned upon. One participant longed for bold and insightful research proposals, which they acknowledged were prone to failure, but felt that the way the funder’s peer-review system was set up did little to stimulate innovation.

I feel that one of the problems with [the funder’s] applications is… the way that the refereeing system is actually structured. It doesn’t build in that question of excitement, at all. So, it’s looking for feasibility and it’s looking for, you know, a judgement about the candidate, a judgement about output, a judgement about judgement. With no sense of, like, what is the most exciting thing you have found about this proposal… it’s quite a good system for extinguishing the spark, really… People are aware of the rules, the funders are aware of the rules. And, what you’re always looking for is somebody who can… turn the game from something routine into something that, kind of, lifts you and actually gives you a moment of insight. And, very rarely it does happen… The most interesting and original work often fails because it has vulnerabilities… by the time something goes through a few referees and the panel… you’re always going to end up with the middle opinion, the middle ground (P3)

Here participants’ quandary was whether to support original but imperfect applications or to err on the side of caution and prioritise the viability of the proposed research. In our sample, familiarity with the applicant appeared to increase participants’ tolerance to risk.

## Discussion

The overall aim of this paper was to gain insights into the experiences of external peer reviewers engaging in the act of evaluating a grant application. This study is the first to examine how independent reviewers experience the evaluation process from a phenomenological standpoint, and the horizons and background assumptions that shape their choices, when assessing an application in near real time.

Our analysis highlighted that reviewers encountered different dilemmas during their evaluation. They were conflicted about whether or not to take on a review, to supplement the contents of the application with pre-existing or new information, to consider institutional prestige, to provide feedback about aspects that are unrelated to one’s area of expertise, and whether to adopt a prudent approach or to favour bold but risky applications. When faced with these dilemmas, reviewers often strove to make the ‘right’ choice by engaging in a series of deliberations and tradeoffs that varied in length, effort and complexity, depending on the reviewer and the dilemma. Peer reviewers’ interpretation of what was right, however, differed and was often influenced by their values, preferences, and experiences. They at times also distanced themselves from relevant norms and their understanding of the funder’s guidelines and priorities, when, for example, they deplored what the funder’s form was not “looking for” (P3), commented on the “nonsense about public engagement” (P6), or admitted putting less emphasis on “the money side of things” (P15). This finding points to the potential usefulness of the concept of *interpretive flexibility* to better understand the peer-reviewing experience. The social constructivist approach to science and technology studies introduced this concept to account for the different interpretations of a given artefact that different social groups may have (Pinch and Bijker, 1984). Interpretive flexibility has notably been discussed in relation to *boundary objects* (Doolin and McLeod, 2012; Star, 2010).

In like vein, our findings invite a (re)conceptualisation of peer review as a process (rather than an outcome) as well as a (re) conceptualisation of the grant application, the written reviews, and funder’s criteria documents as boundary objects subject to interpretive flexibility. Boundary objects are “a sort of arrangement that allows different groups to work together without consensus” (Star, 2010, p. 602). They are not synonymous with interpretive flexibility and are “at once material and processual” (ibid.). They maintain a common identity across the various communities of practice involved in the review process (external reviewers from different fields, panel members, funders), yet they may also be interpreted, understood, and used differently across stakeholders and settings. These boundary objects support cooperation and knowledge sharing without necessarily being underpinned by an implicit or explicit consensus on their usage, meaning or interpretation. This provides an interesting novel perspective, calling for more research on the “invisible work” of peer review, to paraphrase Star (2010). She illustrated how boundary objects may have different meanings in different spaces. In one of her examples, she identified the report of an experiment as a boundary object, which may be interpreted differently by the scientist who conducted the experiment and its peers who later read it:

I turned to one experiment where Ferrier records his attempt at trying to measure the effect of a lesion he produced earlier in the day, on the brain of an ape. The ape is less than cooperative. Ferrier’s handwriting occasionally flies off the page, wobbles, and trails off in what clearly is a chase around the room after the hapless animal. The pages, in sharp contrast to my chapel-like surrounds, are stained with blood, tissue preservative, and other undocumented fluids. By contrast–and this is a finding repeated in sociology of science through the 1980s–the report of the experiment is clean, deleting mention of the vicissitudes of this experimental setting. (p. 606).

Similarly, our results and cognitive process-tracing approach led us to catch a glimpse of another kind of “invisible” work: peer reviewers engaging with their reviewing task. This process involved dilemmatic considerations, led some to wander outside the proposal to seek additional information. This extraneous activity and dilemmatic considerations, however, remain invisible and sanitised out of the reviews’ final orderly ratings and brief comments. Making this invisible work more visible through future research will allows us to better understand why expert reviewers’ decisions can be idiosyncratic and sometimes diametrically opposed to each other’s. The dilemmas we have uncovered suggest that peer reviewers engage in thoughtful considerations during the peer-review process. We should, therefore, be wary of reducing the absence of consensus as resulting from biased, intuitive thinking. Rather, these findings highlight the diverse horizons and background assumption each reviewer brings on the applicants and their project proposals. The processes around how the proposals are used to form peer-review judgements are still ill-defined: the starting position of the review process and the allowable activities during the review are underspecified, and a unique consensual judgement outcome cannot be shown to exist. From this perspective, we can embrace the diversity of opinions as a bonus rather than a bias (Page, 2017). These different points of view and approaches can be particularly enriching during deliberations in a panel setting, where rules are collectively negotiated and enforced (Lamont, 2009).

This being acknowledged, our results also suggest that reviewers’ quandaries started with the decision to take on a review and that acceptance was underpinned by different and sometimes multiple motivations, suggesting that these processes are currently ill-defined and likely discipline specific. Although some of these motivations were relevant and to be expected, such as expertise and interest in the topic, others, such as knowing the applicant, could unduly influence the approach reviewers employ during the evaluation process.

Knowing applicants was indeed a commonly raised concern, and while participants attempted to ‘play by the rules’ by, for example, disclosing this knowledge to the funder, they chose the path that was most consistent with their beliefs. Participants for whom impartiality was paramount, refrained from seeking additional information or voluntarily engaged in a form of blind review, by first reading the scientific content of the application. In contrast, those who felt it was inevitable (and perhaps desirable) to know applicants, had few qualms about using additional information about their track-record in their evaluation. These differences could lead to variation in peer-review outcomes, and favour applicants who are known to reviewers (Li and Agha, 2015; Marsh et al., 2007; Sandström and Hällsten, 2008). Lamont observed that panel members were frequently torn between selfinterest (e.g., supporting familiar applicants) and evaluation (e.g., rigorously assessing the application) (Lamont, 2009); yet, unlike research panels, lack of consensus among external reviewers on what is and is not acceptable, and the absence of a group of peers who would inhibit self-interest behaviours, can allow such behaviours to thrive (although, arguably, knowing that one’s review will be read by peers on the panel may act as a form of restraint for such behaviours).

Although applicants’ ties with prestigious institutions (including the funder) did not go unnoticed, an interesting finding is the Robin Hood-esque tendency among some reviewers to support *the underdog*. While this motivation may be commendable and beneficial to applicants from under-resourced institutions, it could also be a reaction to persistent inequalities in research funding, which favour elite institutions and their collaborators (Viner et al., 2004). Our results indicate that this problem may be inadvertently reinforced by sections in the application form, such as “Current and recent research funding (including Wellcome Trust grants)”, which could signal the importance of belonging to the funder’s network of awardees.

A reviewer suggested that guidance from the funder placed emphasis on the feasibility of the proposed research, which might prompt reviewers to focus on this aspect, but could also create a perverse incentive for applicants to submit ‘palatable’ but unoriginal proposals. This view offers a plausible explanation for the relatively low-risk threshold observed among participants, particularly when applicants were unknown to them. An alternative and complementary explanation is that the absence of guidance regarding what levels of risk are acceptable to the funder, may have driven reviewers to take a more cautious approach.

Worthy of note is reviewers’ dilemma about whether to comment on aspects they felt lied outside the realm of professional academic expertise. In a highly competitive research culture, overt dissent or disclosure of one’s limitations may be regarded as a risky strategy that could impact peer reviewers’ relationship with the funder. However, feeling obliged to comment on aspects beyond one’s area of expertise can result in the inadequate assessment of research applications, and can be particularly problematic in the case of interdisciplinary or multidisciplinary research proposals (Langfeldt, 2006).

Although plausible and perhaps unsurprising to those who have had anecdotal experience of peer-reviewing, the identified dilemmas do not fit neatly into pre-existing conceptualisations. They could, however, be characterised as moral conflicts with social considerations. March’s “logic of appropriateness” framework (March, 1994), as conceptualised by Weber and colleagues (Weber et al., 2004), is a useful approach to make sense of dilemmatic decision-making, and social dilemmas in particular. March posited that decisions are shaped by individuals’ (1) situational recognition—the ability to find similarities between a given situation and other situations that are partly or wholly familiar—(2) their identity (e.g. values, traits) and (3) the application of implicit or explicit rules as a means to simplify choices. The appropriateness framework conceives a decision as the answer to the question “What does a person like me (identity) do (rules) in a situation like this (situational recognition)?” (March, 1994; Messick, 1999), which is governed by a logic of “appropriateness” (i.e. determining the appropriate course of action). The ‘situation’, however, is inevitably viewed through the lens of the decision-maker, whose identity will have a bearing on their understanding of said situation and, in turn, their choice of relevant rules, particularly in the absence of explicit guidance (Weber et al., 2004).

Consistent with the appropriateness framework, our results suggest that the way reviewers appraised and dealt with the identified dilemmas was influenced by aspects related to their identity, as exemplified by the quandary of whether or not to consider information beyond the application. Participants’ understanding (situational recognition) of the dilemma appeared to differ according to their values; some participants conceived the dilemma as ‘unfair versus fair evaluation’, whereas others viewed it as ‘less informed versus more informed evaluation’. Consequently, the first group used the heuristic rule ‘Googling applicants is inappropriate’ or ‘knowing who the applicant is can bias the assessment of the application’ to inform their chosen course of action: ‘only evaluate what is “on the page”‘ or ‘assess the research proposal before looking the applicant’s information’, respectively. In contrast, the second group used the funder’s (explicit) rule ‘disclose a potential conflict of interest’—if the applicant was known to the reviewer—or the heuristic rule ‘gather more information about the applicant’—if they were unknown to the reviewer—to inform their course of action: ‘use prior knowledge about the applicant’ or ‘Google them’, respectively. This example illustrates the impact reviewers’ personal characteristics can have on the path they take and underscores how a lack of shared understanding about what is appropriate can result in divergent positions. It also reveals a deficit of explicit rules, which may have left reviewers with little choice but to rely on heuristic ones. Although modifying individual characteristics—such as fair-mindedness—may be unrealistic, the provision of clear and specific guidance could help reviewers to make choices that are more aligned with the ethos and objectives of the funder.

The peer review system is far from perfect, and we echo Lamont’s call to improve our understanding of grant evaluation and its shortcomings to help peer reviewers to carry it out with greater self-awareness (Lamont, 2009), but also to allow funders to harness its benefits (e.g., expertise, diversity of thought) and mitigate its disadvantages (e.g., bias).

### Implications for funding policy and practice

Funders certainly recognise the value of interpretive flexibility and cognitive diversity in peer-reviews, since they call upon expert reviewers’ sense of what constitute an excellent application rather than impose strict criteria, which would reduce peer review to a “boxticking exercise.” Yet, relying only on a couple of peer reviews for a given application is unlikely to be enough to get a valid and reliable judgement of the quality of such application (see e.g., Forscher et al., 2019). At the same time, peer review is a timeconsuming task and peer reviewers’ efforts are not always fully appreciated. While increasing the number of qualitative reviews per application may improve the odds of developing a robust and valid understanding of an application’s quality, it is not feasible in practice. Our analysis, however, points to clear alternative opportunities to improve how reviewers experience the grant evaluation process, and the fairness and trustworthiness of such process. One improvement initiative could be the development of a set of rules—coupled with clear definitions and training—to guide reviewers’ decisions when dealing with typical dilemmas. This set of rules could include guidance about: (1) acceptable ‘uncertainty’ thresholds—complemented with training modules with realistic scenarios—to assist reviewers in determining when it is appropriate to support risky but potentially valuable research; (2) the appropriateness of seeking further information beyond the application; and (3) the funder’s expectations regarding when it is appropriate to withhold or limit feedback. This guidance could be presented as an “agreement of understanding” specifying reviewers’ rights and responsibilities, since formal commitments are more likely to be reflected upon and adhered to (Rousseau, 1995).

Familiarity bias can affect the integrity and legitimacy of grant evaluation (Sandström and Hällsten, 2008). The disclosure of potential conflict of interest, however, may not only be insufficient to curtail it, but could also provide cover for it (O’Neill, 2002). Funders could, therefore, consider ‘shielding’ reviewers from potential bias, by asking them to evaluate the merit of a research proposal without (or before) having access to information about the applicant, including their host institution.

There is also a case for a thorough evaluation of the application and review forms. Thought should be given to the order in which the information is solicited, as it could inadvertently signal importance. Equally, funders could assess the relevance (and unwelcome effects) of some requirements: is asking about applicants’ prior grants from the funder essential? And could it discourage scholars who have not received such grants from applying or encourage applicants to prioritise collaborations with previous awardees? Should peer reviewers be asked to comment on research budgets, as opposed to expert project managers? And should they comment on public engagement or should it be the remit of communication experts?

Inevitably, revisiting the peer-review guidance and processes, as we suggest above, comes with several challenges. Guidance on ethical dilemmas in peer-reviewing grant proposals will undoubtedly be difficult to formulate and will only be of value if they represent a true consensus among all stakeholders and attracts a substantial number of signatories. This is not impossible, however, as initiatives such as the Committee on Publication Ethics (COPE) demonstrate. It would, of course, still be up to peer reviewers to abide by such guidelines but, again, this is not different from other ethical obligations, which already bind professional academic activities such as research or publication ethics. Similarly, redacting grant applicant identifiers may not eliminate reviewers’ ability to second-guess applicants’ characteristics, but there is evidence to suggest that it could reduce halo effects and improve fairness (Nakamura et al., 2021). Overall, therefore, it could be argued that a more effective and equitable peer-review system requires funders, applicants and peer reviewers themselves to collectively agree on what values should be prioritised—which may vary across grant schemes—and what courses of action better enshrine those values.

### Limitations

Our sample, although modest, included reviewers with different levels of experience and from a range of disciplines within the medical humanities and social sciences, who evaluated applications submitted to different funding schemes. Although sexes were fairly distributed within the original sample of 44 reviewers (50% female), women were underrepresented in the final sample of interviewees (25% female) while male participants, those from a White ethnic background, in their 50s or specialising in history, were overrepresented. We do not have any explanation for this other than the fact that our initial decision to advertise the study after the end of the Wellcome Trust July 2018 round of reviews, to reduce recall bias, could have inadvertently prevented some women with childcare responsibilities to take part, due to summer closures of schools and childcare settings. Future studies could test the generalisability of our findings among larger and more balanced samples, and across different disciplines and research funders.

A possible shortcoming is that participants may have engaged in post hoc rationalisation when reviewing the application a second time. However, our primary concern was to avoid adulterating the original review process; thus, this potential limitation was deemed acceptable. Given the sensitive nature of some of the discussed topics, it is also plausible that social desirability bias could have prevented some reviewers from disclosing perceptions or behaviours that were considered inappropriate.

Finally, we could have possibly found evidence for these dilemmas through more standard qualitative interviews without the need to engage reviewers through revisiting a specific grant application via a “thinking aloud” protocol. Yet, we found that remote interviewing using the think aloud approach helped participants to be candid about sensitive issues, to the extent that some asked for reassurance regarding confidentiality, which indicates good rapport. Future research may benefit from further exploring whether our methodology can indeed allow researchers to reduce desirability bias.

## Conclusions

The importance of this study lies in the approach used to elicit reviewers’ experiences, which allowed us to catch a rare glimpse into how independent reviewers grapple with dilemmatic decisions they encounter when evaluating a grant application. This research increases our knowledge on how reviewers’ identity, situational recognition and application of (often heuristic) rules influence their course of action. It also underscores the need to better understand the ‘hidden’ socio-psychological aspects, norms and idiosyncratic practices that shape the peer-review process. If we are to improve peer review, we need to move away from a focus on the individual flaws of peer reviewers and toward an evidence-based and consensus-driven system that makes the best use of reviewers’ expertise, and fosters subjectivity when it is warranted and prevents it when it is not.

## Figures and Tables

**Table 1:** Participant characteristics.

Characteristic	Number in sample (number in population)
^a^Age	
30-39	1 (1)
40-49	3 (16)
50-59	9 (14)
60-69	2 (7)
70+	1 (1)
Sex	
Female	4 (22)
Male	12 (19)
Prefer not to say	0 (3)
^a^Ethnicity	
White	15 (34)
Non-white	1 (3)
Location	
London	2 (5)
North	6 (15)
South	2 (7)
Scotland	2 (3)
Wales	1 (4)
International	3 (7)
Midlands	0 (2)
N. Ireland	0 (1)
Discipline	
History	10 (22)
Sociology	2 (7)
Ethics, Ethics & Law	1 (2)
Philosophy	1 (4)
Economics	1 (1)
Anthropology	1 (1)
English	0 (2)
Public Health	0 (3)
Current role	
Lecturer	5 (unknown)
Reader	3 (unknown)
Professor	8 (unknown)
General peer review experience	
Less than 10 grant applications	4 (unknown)
Between 10 and 20 grant applications	1 (unknown)
More than 20 grant applications	11 (unknown)
Type of award reviewed in this study	
Research fellowship	6 (unknown)
Collaborative award	6 (unknown)
University award	2 (unknown)
Research award for health professionals	2 (unknown)

a The age (*N* = 5) and ethnicity (*N* = 7) of some of the 28 reviewers who did not participate in this study were unknown.

## Data Availability

Upon completion of the TORR project, pseudonymised transcripts and general participant characteristics (e.g., gender, country of residence) will be deposited in the UK Data Archive (http://data-archive.ac.uk) and may be shared with other researchers on reasonable request, in compliance with the Wellcome Trust’s policy on data, software and materials management and sharing.
